# Effectiveness of “Palliative Care Information Booklet” in Enhancing Nurses’ Knowledge

**DOI:** 10.4103/0973-1075.73647

**Published:** 2010

**Authors:** Anita David, Sonali Banerjee

**Affiliations:** Department of Medical Surgical Nursing, Apollo College of Nursing, Apollo Health City, Jubilee Hills, Hyderabad, India

**Keywords:** Information booklet regarding palliative care, Knowledge of nurses, Advanced disease

## Abstract

**Context:**

Patients diagnosed with a disease like cancer require not only physical control of disease but also they need psychological reassurance, social and spiritual support in coming to terms with their disease. Nurses working in the specialized cancer hospitals play a significant role in the care of terminally ill patients. They must be knowledgeable, skilled and sensitive to the needs of these patients and their families in order to provide active, holistic care.

**Aims:**

In this study, we attempted to assess the level of knowledge about palliative care among nurses working in the oncology department using a self administered structured questionnaire and also to assess the effectiveness of information booklet designed on various aspects of palliative care on their knowledge.

**Settings and Design:**

Indo American Cancer Hospital, Hyderabad, AP, India. The design adopted for this study was One Group pretest – posttest, pre - experimental design.

**Materials and Methods:**

Hundred nurses working in Indo American Cancer Hospital, Hyderabad, AP, India were selected by using the non probability purposive sampling technique. A structured self administered questionnaire was prepared and administered as a pretest. An information booklet was developed pertaining to the general concepts of palliative care, care components (physical, social, emotional and spiritual) and role of the nurse in palliative care and it was given to the participants. As a post test, the same questionnaire was re-administered after four days to the same study subjects. Pretest and post test knowledge scores were compared and the findings were analyzed statistically.

**Statistical analysis used:**

Microsoft Excel and Statistical Package for Social Science package.

**Results:**

The post test scores were significantly higher than the pretest knowledge scores, which indicate that the developed information booklet regarding palliative care was highly effective in enhancing the knowledge levels of the nurses.

**Conclusions:**

The information booklet was effective in enriching the knowledge of nurses on palliative care. Enhancing the nurse’s knowledge about palliative care will promote their understanding of the needs of the advanced stage patients and will enable them to provide quality care.

## INTRODUCTION

Palliative care had been a much-ignored field but now people are gaining more awareness and are giving it more importance because of the increasing number of people with terminal illness[1]. Cancer is a major cause of morbidity in India. It was estimated that 2.5 million people are diagnosed with cancer every year in the country. Of these 2.5 million people, 70.0% of them require palliative care but less than 0.4% is able to access it.

Nurses play a significant role in the care of the dying, critically ill as well as the terminally ill clients. Lack of knowledge about palliative care is an obstacle to nurses and health care professionals as they strive to deliver palliative care. The ultimate goal is to improve palliative care for patients in all settings and enhance the experience of family members witnessing the dying process of their loved ones.[2] Nurses will be able to render effective palliative care when they are able to identify the symptoms and the needs of the patient. Nakazawa Y., Miyashita M., Morita T.,[3] conducted a study in Japan to evaluate palliativecare knowledge among general physicians and nurses and to measure the efficacy of palliative care educational programs. The questionnaire was distributed to 940 nurses and 85% of the nurses were not aware of the physical symptoms of the patients that require palliative care. It was concluded that more emphasis should be given on palliative care either at the undergraduate level or through continuing education programs for nurses.

## MATERIALS AND METHODS

Objectives of the study:

1. To assess the level of knowledge about palliative care among nurses working in Indo American Cancer Hospital.
2. To assess the effectiveness of the developed information booklet about palliative care on the level of knowledge of nurses.

The null hypothesis formulated for the study was – “There is no significant difference between pretest and post test knowledge scores of nurses about palliative care”.

A Pre-experimental One Group pretest–post test design was undertaken for the study. It was conducted in the month of April 2010 at Indo American Cancer hospital, which is one of the super specialized cancer hospitals in Hyderabad, Andhra Pradesh. A written permission was taken from the Medical Director and Nursing Superintendent of the Hospital. Target population comprised 180 nurses and purposive sampling technique was used for collecting the sample. Inclusion criteria for the study: nurses working in the oncology wards, cubicles, private rooms, ICU and those who have completed GNM/B.Sc. /PC B.Sc. nursing or specialized in oncology nursing and were present at the time of conducting the pretest. Exclusion criteria: nurses who were off duty on the day of pre-test, nurse assistants who have done ANM course and nurses working in Operation Theater, investigation rooms and radiation department. Sample comprised 100 nurses working inIndo American Cancer hospital.

Conceptual framework of the study was adopted from the Daniel Stufflebeam’s CIPP (Context – Input – Process – Product evaluation) model 2003.[4] An information booklet was developed pertaining to the general concepts of palliative care, care components (physical, social, emotional and spiritual) and role of the nurse in palliative care. A structured self administered questionnaire comprising four sections was developed to gather relevant information from the subjects. section A comprised items related to the demographic data, section B dealt with general knowledge about palliative care, section C dealt with the components of palliative care and section D had questions related to the role of the nurse in palliative care. Eleven experts from the field of nursing, palliative care, medicine, clinical psychology and statistics were chosen to validate the tool and the information booklet. The reliability of the tool was elicited using the Test-Retest method by using Spearman’s rank correlation coefficient. The calculated correlation value (0.98) was found to be significant. Feasibility of the study was tested. Professional ethics were maintained throughout the study.

A pretest in the form of self administered structured questionnaire on palliative care was administered to 100 nurses working at Indo American Cancer hospital. Immediately after the test, an information booklet regarding palliative care was given to each participant. After four days, the same questionnaire was re-administered to the same participants and the knowledge levels before and after giving the information booklet were computed and compared through statistical analysis.

## RESULTS

### Description of sample characteristics

- Majority (64.0%) of the nurses who participated in the study were below 26 years of age, mean age was 26.55±2.15.
- Majority (84.0%) of the nurses had done General nursing and Midwifery, 12.0 % of them were BSc nurses and 4.0% had completed PC BSc nursing.
- A majority (84.0%) of the subjects did not have palliative care included in their nursing curriculum.
- Majority (46.0%) of the nurses had work experience of less than two years in oncology department and 31% of the nurses had about two–four years of work experience. Eight percent had four–six years of experience and only 15% of the nurses had more than six years of experience.
- Out of the 100 nurses only eight of them received specialized training in palliative care.

### Assessing effectiveness of information booklet regarding palliative care

Effectiveness of the information booklet was assessed by using Chi square test and paired ‘t’ test.

When comparing the knowledge scores, the post test knowledge scores were significantly higher than the pretest scores for each section of the questionnaire. The calculated Chi square values (2 df, *P* < 0.05) were found to be significant (tabulated χ^2^ =5.999, 2df, *P*=0.000)

- The mean post test score was 21.78±3.46 which showed an increase of 9.07 as compared to the mean of pretest scores which was 12.71±3.13. The computed ‘*t*’ value at 99df, *P* < 0.05 level of significance was 19.77 which was higher than the tabulated ‘*t*’ value (1.699) [Table 1].
- This difference is statistically significant; hence, the framed hypothesis that “there is no significant difference between the pre-test and post-test knowledge scores of nurses on palliative care” was rejected. This confirmed that the information booklet that was developed and used in the study was effective in increasing the knowledge regarding palliative care among nurses working in Indo American Cancer Hospital.

**Table 1:** Comparison of pretest and post-test knowledge scores using paired ‘*t*’ test n = 100

Test	Freq.	Mean	S D	Mean difference	‘*t*’ value df = 99
Pre test	100	12.71	3.13	9.07	19.77
Post test	100	21.78	3.46		*P* < 0.05

## DISCUSSION

No similar study has been reported in India; various reviews are available from studies conducted in western countries.

### Demographic characteristics of the nurses who participated in the study

**Figure 1:**
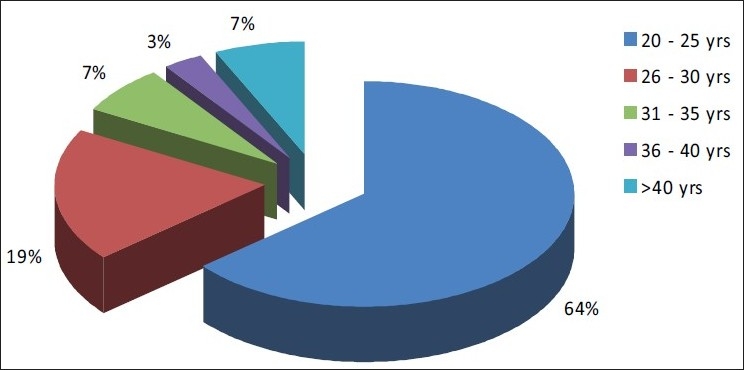
Pie chart showing the percentage distribution of nurses based on their age

Majority (64.0%) of the nurses who participated in the study were below 26 years of age and mean age was 26.55±2.15 [Figure 1]. Margaret *et al*.[5] conducted a study on nurses’ knowledge on palliative care and reported that majority (76.0%) of the nurses who participated in the study were between 25 and 40 years of age. Educating the young graduates or providing ongoing education to them on palliative care not only generates awareness about palliative care but also it enhances their knowledge on the special needs of the patient and the family and enables her to provide need-based care.

A majority (84.0%) of the nurses did not have palliative care included in their study curriculum. Only 16 of them had palliative care included in their nursing curriculum. This was reflected in the pretest scores, mean value 12.71±3.13 which was very low as compared with the post test results, which was conducted after giving the information booklet. Ferrell BR, *et al*.[6] conducted a study on the inadequacies of nursing education and pointed out that nursing subjects have limited content on end of life care. It was reported that increased attention to this area will significantly enhance the nurse’s knowledge and competency in providing quality end-of-life care for the terminally ill. Introducing palliative care in the nursing curriculum will play a vital role in equipping the nurse with the needed knowledge to work efficiently with terminally ill patients.

Out of the 100 nurses, only eight of them received specialized training in palliative care. This could probably be related to the fact that majority (46.0%) of the nurses had less than two years of experience and 84.0% of them did not have palliative care in their nursing curriculum. Abu-Saad Huijer H, *et al*[7] in their study on the knowledge, attitude and practices of medical and nursing specialties concluded a positive association between practice scores and continuing education. It was suggested that formal education in palliative care enhances the competency of the nurse in providing holistic care to the terminally ill patients. The competency of nurses working in oncology hospitals or in other health care setups for terminally ill patients can be enhanced through specialized formal education or continuing education on palliative care.

### Assessing effectiveness of information booklet on palliative care

Chi square was computed to show the effectiveness of the information booklet. In all the three sections of the questionnaire, the post test scores were significantly higher than the pretest scores. The calculated Chi square values Table 2 to show the comparison between pretest and posttest at 2df, *P* <0.05 level of significance were 87.928 in section B, 83.176 in section C and 127.77 in section D while the tabulated value was 5.991. This confirmed that the information booklet was effective in improving the knowledge of the nurses about palliative care. The mean of the posttest score showed an increase of 9.07 which is significantly higher than the mean pretest knowledge scores [Table 1] at 0.05 level of significance. The computed ‘*t*’ value was 19.77 at 2df *p*<0.05 level of significance [Table 1] indicated a statistical significance and confirmed the effectiveness of the information booklet. In a study conducted by Knapp CA *et al*.,[8] it was reported that there was a significant gain of knowledge after providing education material to nurses working in oncology department. The findings of the study are in congruence with the present study and hence it was suggested that providing information booklet is an effective strategy to increase the knowledge of nurses.

**Table 2:** Comparison between the pretest and post test knowledge scores as per the three sections in the questionnaire n=100

Sections of the questionnaire		Below average (</=50%)	Average (51-75%)	Above average (>75%)	Total	χ^2^	df	*P* value	S/NS
Section B	Pre test	86	10	4	100	87.928	2	.000	S
	Post test	21	29	50	100				
Section C	Pre test	65	33	2	100	83.176	2	.000	S
	Post test	11	39	50	100				
Section D	Pre test	92	8	0	100	127.77	2	.000	S
	Post test	13	40	47	100				

S – Significant; NS – Not significant

## CONCLUSION

Knowledge provides an organized body of information that is factual; it provides a foundation of correct principles and concepts. Application of this knowledge develops and enhances nursing skills. Nurses play an important role in the delivery of palliative care services. A nurse who appreciates her role in patient care will take the responsibility to equip herself with necessary knowledge to upgrade her skills and put them to the best use. It will also enable her to assess individual situations, integrate those experiences with the knowledge and render quality health care. A variety of teaching methods and strategies and ongoing or continuing education programs can be used to ensure the best possible learning experience. The present study has been designed to determine the effectiveness of the developed information booklet about palliative care on the level of knowledge of nurses. The intervention in the form of information booklet about palliative care had shown that there has been a significant improvement in the knowledge level of the participants. Hence it can be concluded that providing an information booklet is an effective method in enhancing the knowledge level of nurses.
